# Transplantation of insulin-producing cells from umbilical cord mesenchymal stem cells for the treatment of streptozotocin-induced diabetic rats

**DOI:** 10.1186/1423-0127-19-47

**Published:** 2012-04-30

**Authors:** Pei-Jiun Tsai, Hwai-Shi Wang, Yi-Ming Shyr, Zen-Chung Weng, Ling-Chen Tai, Jia-Fwu Shyu, Tien-Hua Chen

**Affiliations:** 1 Institute of Clinical Medicine, National Yang-Ming University, Taipei, Republic of China; 2 Department of Emergency, Division of Surgery, Veteran General Hospital, Taipei, Republic of China; 3 Institute of Anatomy and Cell Biology, School of Medicine, National Yang Ming University, 201 Shih-Pai Road Section 2, Taipei, 112, Republic of China; 4 Department of Surgery, Veteran General Hospital, Taipei, Republic of China; 5 Division of Cardiovascular Surgery, Cardiovascular Center, Taipei Medical University Hospital, Taipei, Republic of China; 6 Department of Biology and Anatomy, National Defense Medical Center, 161 Ming Chuan E. Road Section 6, Taipei, 114, Republic of China

**Keywords:** Mesenchymal stem cell, Portal vein, Insulin-producing cells, Transplant

## Abstract

**Background:**

Although diabetes mellitus (DM) can be treated with islet transplantation, a scarcity of donors limits the utility of this technique. This study investigated whether human mesenchymal stem cells (MSCs) from umbilical cord could be induced efficiently to differentiate into insulin-producing cells. Secondly, we evaluated the effect of portal vein transplantation of these differentiated cells in the treatment of streptozotocin-induced diabetes in rats.

**Methods:**

MSCs from human umbilical cord were induced in three stages to differentiate into insulin-producing cells and evaluated by immunocytochemistry, reverse transcriptase, and real-time PCR, and ELISA. Differentiated cells were transplanted into the liver of diabetic rats using a Port-A catheter via the portal vein. Blood glucose levels were monitored weekly.

**Results:**

Human nuclei and C-peptide were detected in the rat liver by immunohistochemistry. Pancreatic β-cell development-related genes were expressed in the differentiated cells. C-peptide release was increased after glucose challenge *in vitro*. Furthermore, after transplantation of differentiated cells into the diabetic rats, blood sugar level decreased. Insulin-producing cells containing human C-peptide and human nuclei were located in the liver.

**Conclusion:**

Thus, a Port-A catheter can be used to transplant differentiated insulin-producing cells from human MSCs into the portal vein to alleviate hyperglycemia among diabetic rats.

## Background

Type 1 DM is an autoimmune disease that is characterized by inhibited insulin production as a result of T cell-mediated destruction of the pancreatic β cells in the islets of Langerhans [1,2]. Transplantation therapies for type 1 DM include whole organ transplantation [3], transplantation of isolated islets [4,5] and regeneration therapy [6]. Although the transplantation of both a whole organ and isolated islets has been successfully used in the clinical treatment of type 1 DM, a shortage of donors limits the widespread use of this treatment modality. Additionally, the quality of a donor’s pancreas is an important criterion for islet isolation [7]. On the other hand, regeneration therapy, in which stem cells are stimulated to differentiate into insulin-producing cells that can be used to replace lost β cells, is free of such supply limitations [8].

Mesenchymal stem cells (MSCs) were first isolated from bone marrow [9] and have the potential to differentiate in culture into muscle cells, adipocytes, osteocytes, chondrocytes [10-12], cardiomyocytes [13-16] and pancreatic β cells [17]. Moreover, following systemic injection, MSCs have been shown to be incorporated into a variety of tissues, including bone [18,19], muscle [20], lung [21] and epithelium [22]. Although insulin-producing cells can be developed from bone marrow MSCs [23], adipose tissue-derived stem cells [24], and human umbilical cord blood-derived mononuclear cells [25], the number of MSCs that can be cost-effectively isolated and differentiated remains a major limitation. We found that fibroblast-like cells from Wharton’s jelly of the human umbilical cord were similar to MSCs in the bone marrow and could be induced to differentiate into adipogenic cells, osteogenic cells, cardiomyogenic cells, and insulin-producing cells [26,27]. Because MSCs from the umbilical cord can be easily isolated and expanded in culture, they may provide a novel source of cells for cellular type 1 DM therapies.

In this study, we first characterized the insulin-producing cells derived from MSCs of Wharton’s jelly with modified three stages β cell differentiation method [17]. Subsequently, we treated diabetic rats by transplanting the differentiated insulin-producing cells into their livers through the portal vein. Instead of using renal subcapsular space [28] or tail vein [29] transplantation, we used a specially designed Port-A catheter portal delivery system that has been used in human islets transplantation [4,5]. In this study, streptozotocin (STZ) was used to induce type 1 DM because there is extensive evidence that hyperglycemia induced by STZ can be lowered by stem cell therapy [30-33]. STZ is a naturally occurring chemical that is toxic to pancreatic β-cells in mammals and can produce an animal model of type 1 DM. The aim of this study was to test the curative effect of transplanting insulin-producing cells differentiated from human Wharton’s jelly MSCs into rat livers.

## Methods

### Cell culture

Institutional Review Board approval was obtained for all procedures. With the written informed consent of the parents, fresh human umbilical cords were obtained after birth and stored in Hank’s balanced salt solution (Biological Industries, Israel) prior to tissue processing to obtain MSCs. The isolation of MSCs followed the methods set forth by Wang et al. [26]. Briefly, after removal of blood vessels, the mesenchymal tissue was scraped off the Wharton’s jelly and centrifuged at 250 *g* for 5 min. After centrifugation, the pellets were re-suspended in 15 ml of serum-free Dulbecco’s modified Eagle’s medium (DMEM; Gibco, Grand Island, NY) containing 0.2 g/ml of collagenase and incubated for 16 h at 37°C. Next, the cells were washed, resuspended in DMEM containing 2.5% trypsin, and incubated for 30 min at 37°C with agitation. Finally, cells were again washed, and cultured in DMEM supplemented with 10% fetal bovine serum (FBS; Sigma St. Louis, MO, USA) and glucose (4.5 g/l) in 5% CO_2_ in a 37°C incubator.

### *In vitro* differentiation cultures

**Figure 1:**
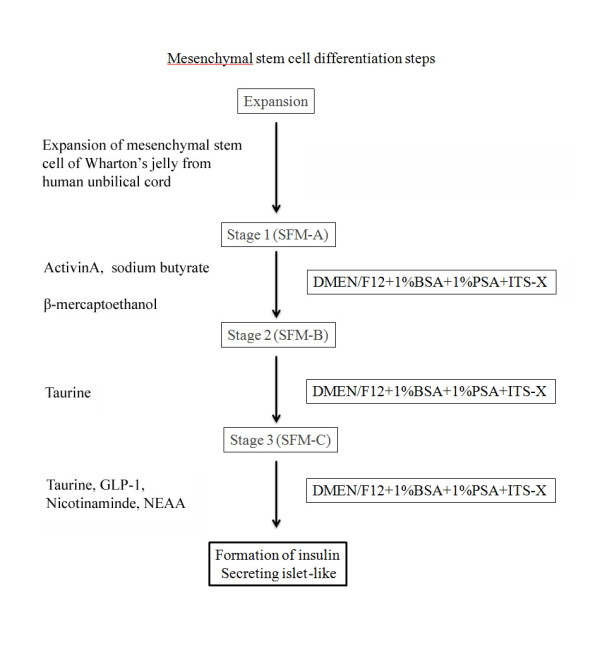
Diffenentiation scheme for generating insulin-producing cells from human umbilical cord mesenchymal stem cells.

Differentiation was carried out in three stages, as shown in Figure 1. At the fourth to sixth passage, after reaching a confluence of 70% the MSCs were induced to differentiate into islet-like cell aggregates. Differentiation was divided into three stages. Undifferentiated human mesenchymal stem cells (HUMSCs) were detached by HyQTase, diluted with SFM-A and centrifuged. Cells were counted for initial seeding density and 1 10^6^ cells/cm^2^ were resuspended in SFM-A and seeded on ultralow attachment tissue culture plates (Corning, Fisher Scientific International, Hampton, NH, http://www.fisherscientific.com). SFM-A contained DMEM/F12 (1:1) (Gibco, Grand Island, NY) with 17.5 mM glucose, 1% BSA Cohn fraction V, fatty acid free (Sigma-Aldrich), 1% penicillin/streptomycin/amphoteric B (PSA; Biological Industries, Israel), 1X insulin-transferrin-selenium-X (ITS-X; 5 mg/l insulin, 5 mg/l transferrin, 5 mg/l selenium), 4 nM activin A, 1 mM sodium butyrate, and 50 μM 2-mercaptoethanol. The cells were cultured in this medium for 2 days. On the third day, the culture medium was changed to SFM-B, which contains DMEM/F12 (1:1) with 17.5 mM glucose, 1% BSA, 1%PSA, ITS-X, and 0.3 mM taurine. On the fifth day, the cell culture was replaced by SFM-C, which contained DMEM/F12 (1:1) with 17.5 mM glucose, 1.5% BSA, ITS-X, 1%PSA, 3 mM taurine, 100 nM glucagon-like peptide (GLP)-1 (amide fragment 7–36; Sigma Aldrich), 1 mM nicotinamide, and 1X nonessential amino acids (NEAAs). For the next 5 days, the culture medium was exchanged with fresh SFM-C every 2 days [24]. The differentiation condition was modified from that previously established [27], and lasted for a total of 10 days.

### Immunocytochemical staining for C-peptide

Control and differentiated cells on coverslips were fixed with 4% paraformaldehyde for 15 minutes, and washed with phosphate-buffered saline (PBS). A DakoCytomation EnVision+Dual system-HRP kit (DakoCytomation Inc, CA) and mouse anti-human C-peptide antibody (Chemicon, Billerica, MA) were used to stain the cells. Briefly, Dual Endogenous Enzyme Block solution was added to cover the coverslips for 10 minutes. Next, the cells were washed with PBS and incubated for 1 h at 37°C with mouse anti-human C-peptide monoclonal antibody (1:100). After washing with PBS, the coverslips were incubated for 30 min with labeled Polymer-HRP. After another round of washing with PBS, Substrate-Chromogen was added for 5 min. Finally, the coverslips were washed with distilled water. Cell nuclei were visualized by incubating the coverslips for 5 min at room temperature with hematoxylin (Sigma Aldrich, St. Louis).

### Reverse transcriptase-polymerase chain reaction (PCR) analysis

Total RNA was extracted from control and differentiated cells using RNeasy Purification Reagent (Qiagen, Valencia, CA). Subsequently, a 4 μg sample was reverse transcribed with Mmlv reverse transcriptase (Amersham, Uppsala, Sweden) for 30 min at 42°C in the presence of an oligo-dT primer. The PCR reaction mixture consisted of 38.5 μl of sterile distilled water, 5 μl of 10X PCR buffer, 1 μl of dNTP, 1.5 μl of each primer, 2 μl of cDNA (4 μg), and 0.5 μl of polymerase (5 U**/**μl) (Amersham). cDNA was amplified for 30 cycles (94°C for 45 s, annealing for 45 s, and 72°C for 40 s) using the following primer sequences: *Pdx1* forward GGAGCCGGAGGAGAACAAG, reverse CTCGGTCAAGTTCAACATGACAG; *Pax4* forward GGGTCTGGTTTTCCAACAGAAG, reverse CAGCGCTGCTGGACTT; *insulin*, forward GCTGGTAGAGGGAGCAGATG, reverse AGCCTTTGTGAACCAACACC; GAPDH, forward CACCATCTTCCAGGAGCGAG, reverse TCACGCCACAGTTTCCCGGA (Mission Biotech, Taiwan). PCR was performed for 30 cycles of denaturation at 95°C for 30 s, annealing at 55-63°C for 30 s, and elongation at 72°C for 1 min, with a final 10 min extension at 72°C. To exclude the possibility of contaminating genomic DNA, PCRs were also run without reverse transcriptase. The amplified cDNA was separated by electrophoresis on a 1% agarose gel, stained, and photographed under ultraviolet light.

### Real-time PCR analysis

cDNA was prepared from 4 μg of total RNA as described above and 200 ng of RNA equivalents was used for PCR with specific primers in the presence of SYBR Green I (Light Cycler^TM^-FastStart DNA Master SYBR Green I; Roche, Basel, Switzerland). The sequences of the primers were as follows: *Pdx1* forward GGAGCCGGAGGAGAACAAG, reverse CTCGGTCAAGTTCAACATGACAG; *Pax4* forward GGGTCTGGTTTTCCAACAGAAG, reverse CAGCGCTGCTGGACTT; *insulin* forward ACCAGCATCTGCTCCCTCTA, reverse GGTTCAAGGGCTTTATTCCA; GAPDH forward CACCATCTTCCAGGAGCGAG, reverse TCACGCCACAGTTTCCCGGA (Mission Biotech). A LightCycler® 480 (Roche, Indianapolis, IN) was used for real-time PCR.

### Measurement of spontaneous C-peptide secretion

After 10 days of differentiation, the cells were washed with PBS and incubated for 3 h in DMEM-LG (5.5 mM glucose) (Gibco, NY). The medium was collected and stored at −20°C until being assayed. C-peptide ELISA kit (Mercodia, Uppsala, Sweden) was used according to the manufacturer’s instructions. Briefly, 25 μl of the sample was added to 50 μl of assay buffer in the wells of a 96-well plate coated with anti-human C-peptide antibody. The mixture was incubated for 1 h at 18-25°C on a shaker. After six washes with washing buffer, 100 μl of enzyme conjugate was added to the mixture and incubated on a shaker for 1 h at 18-25°C. Next, 200 μl of substrate TMB was added for 15 min. Finally, stop solution (50 μl) was added to the wells for 5 sec and the absorbance was read at 450 nm.

### Glucose challenge test

After 10 days of differentiation, the cells were incubated for 1 h in DMEM-LG (5.5 mM glucose), and the medium was collected and stored at −20°C. Next, the cells were washed with PBS and incubated for 1 h in DMEM-HG (25 mM glucose; Gibco, NY) and the medium was collected and stored at −20°C. The C-peptide concentration was determined using the C-peptide ELISA kit.

### Laboratory animals

A total of 18 male, 6- to 8-weeks-old Sprague Dawley rats, weighing between 350–450 g (Laboratory Animal Center, Yang-Ming University, Taiwan) were provided with food and water *ad libitum* and were housed on a 12-h light and 12-h dark cycle. The experiment followed institutional guidelines pertaining to animal welfare.

### Induction of DM with STZ

One week after surgery, 30 mg/kg of STZ ((Sigma Aldrich) solution in acidified 0.9% saline (pH 4.5) was injected intraperitoneally into the rats on 3 consecutive days to induce type 1 DM.

### Transplantation

Rats were divided into one control group (without STZ induction), and two transplantation groups, each of which consisted of six rats. One week after STZ induction, rats the experimental group were restrained and injected with 5 × 10^6^ differentiated insulin-producing cells suspended in 0.1 ml of normal saline, followed by a volume of normal saline equivalent to the volume of the Port-A catheter (0.35 ml) to push the grafts into the portal vein. The control group underwent the same procedure, but was only injected with normal saline (STZ group).

### Physiological monitoring

Body weight and blood sugar were recorded every week after transplantation. Sugar levels in the blood collected from the tail vein were measured using a blood glucose meter (Roche, Indianapolis, IN).

### Immunohistochemistry

Eight weeks following transplantation the rats were sacrificed and perfused with 4% formaldehyde (Ferak, Berlin, Germany). Rat livers were cut into 0.5-1.0 cm^3^ pieces. The samples were dehydrated and embedded in OCT (Sakura Fintek, USA) in liquid nitrogen. The cryosections (5 μm) were washed with PBS, then incubated overnight at 4°C with mouse anti-human nuclei monoclonal antibody (1:400; Chemicon, Billerica, MA), and rabbit anti-human C-peptide antibodies (1:100; Santa Cruz, Santa Cruz, CA). After washing with PBS, the slides were subsequently incubated for 1 h with Cy3-labeled goat anti-human IgG antibodies (1:200) and rhodamine conjugated goat anti-rabbit IgG antibodies (1:500) (both from Chemicon). The sections were mounted with mounting medium (Vector) and viewed on a fluorescence microscope.

### Statistical analysis

Each series of experiments was performed in triplicate. The results obtained from a typical experiment were expressed as the means ± standard deviation (SD). Statistical analysis was carried out using the SPSS 14.0 software program (Statistics Package for Social Sciences, SPSS Inc. Chicago, Illinois, USA). All continuous data were presented as mean ± SD. Student’s *t*-test was used to compare the means of two groups. Categorical variables were compared by χ^2^ test or Fisher’s exact test. A P value of less than, or equal to 0.05 was considered to be statistically significant.

## Results

### Gene expression in the differentiated cells

**Figure 2:**
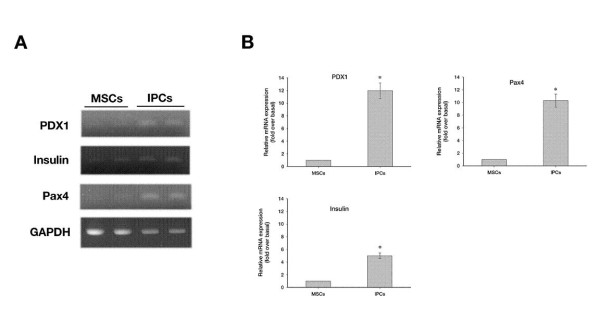
**Reverse transcriptase and real-time PCR analysis of the expression of pancreatic β-cell development-related genes and insulin production-related genes.(A)** Reverse transcriptase -PCR results for non-differentiated cells (MSCs, lane 1 and 2), differentiated cells (IPCs, lane 3 and 4). **(B)** Real-time PCR analysis of the expression of *Pax4*, *Pax1* and *Insulin.* Results are expressed as the mean ± SEM for 4 experiments. *, P ≪ 0.05 compared to the non-differentiated cells.

To determine whether the MSCs had differentiated into insulin-producing cells, the expression of genes involved in pancreatic β-cell development and insulin production was examined by reverse transcriptase-PCR and real-time PCR. As shown in Figure 2, the pancreatic β-cell development-related genes *Pax1*, *Pax4*, and *insulin* was expressed in differentiated medium. However, the expression of *Pax1*, *Pax4*, and *insulin* was significantly greater in differentiated IPCs versus undifferentiated MSCs (Figure 2).

### Detection of C-peptide in the differentiated cells derived from mesenchymal stem cells from the umbilical cord

**Figure 3:**
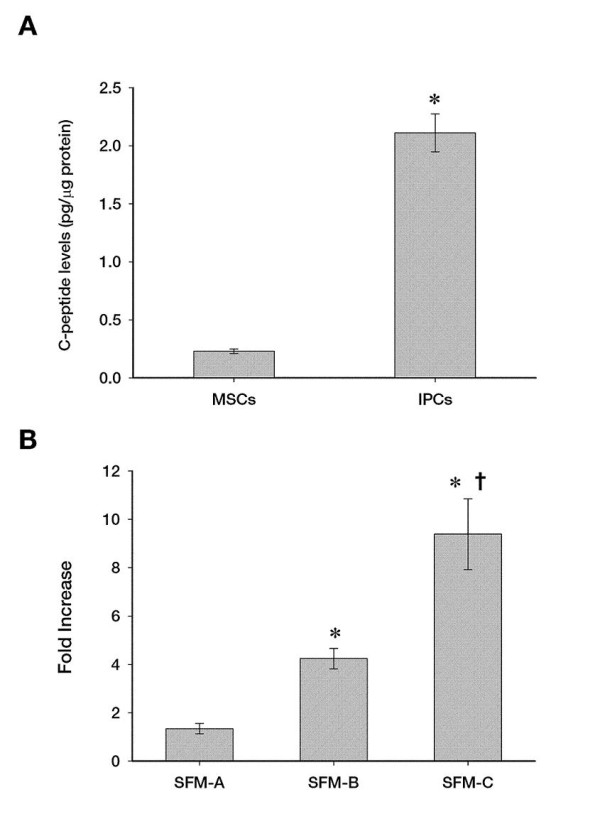
**Measurement of spontaneous C-peptide secretion showing that the mesenchymal stem cells of Wharton’s jelly differentiate into insulin-producing cells.** The expression of C-peptide was greater among differentiated IPCs versus undifferentiated MSCs (Figure 3A). The level of C-peptide expression increased several-fold from MSCs cultured in SFM-A, SFM-B and finally SFM-C (Figure 3B). *, P ≪ 0.05 compared to the non-differentiated cells in A and compared to SFM-A in B. †,P ≪ 0.05 compared to SFM-B.

Anti-human C-peptide antibodies revealed that C-peptide was expressed in undifferentiated MSCs and differentiated IPCs. However, the expression of C-peptide was greater in differentiated IPCsthan in undifferentiated MSCs (Figure 3A). As illustrated in Figure 2B, the expression of C-peptide level increased several-fold from MSCs cultured in SFM-A, SFM-B and finally SFM-C (Figure 3B).

### Immunofluorescent staining for C-peptide

**Figure 4:**
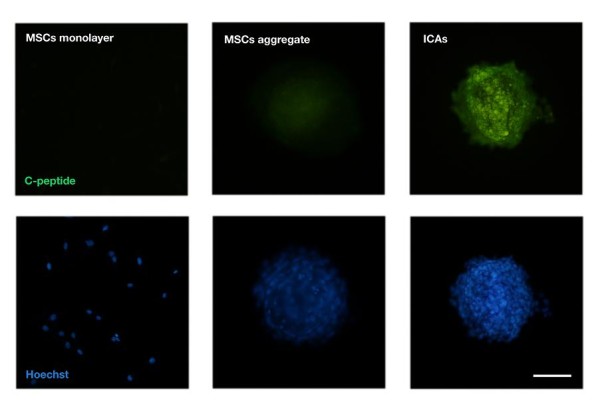
**Immunocytochemical staining for C-peptide.** The MSCs were induced into islet-like cell clusters (ICAs) which contain C-peptide labeling cells, indicating that the induced MSCs could aggregate and form functional pancreatic endocrine cells. Bar = 50 μm.

The results of immunofluorescent analysis of the protein expression in insulin-producing cells cultured in Control, undifferentiated and differentiated MSCs were shown in Figure 4. The Cellular aggregation occurred as a gradual process in undifferentiated MSCs and complete aggregates were formed in differentiated IPCs.

### Secretion of C-peptide by insulin-producing cells upon glucose stimulation

**Figure 5:**
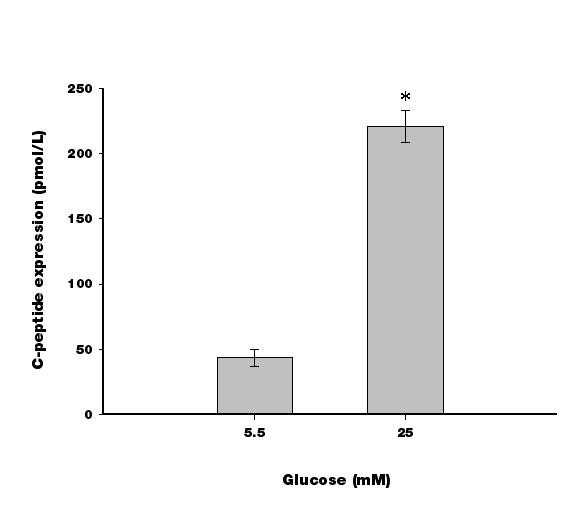
**Glucose challenge test for C-peptide release in response to low (5.5 mM) or high (25 mM) glucose concentrations of differentiated cells.** (*, P ≪ 0.05 compared to 5.5 mM glucose). Results are expressed as the mean ± SEM for 4 experiments.

C-peptide is a byproduct of new insulin formation. The concentration of C-peptide in the undifferentiated and differentiated cells, measured by ELISA, showed that these cells secreted higher levels of C-peptide than the pretreatment cells (Figure 2A). To determine whether these insulin-producing cells were responsive to glucose challenge, C-peptide secretion was measured. As shown in Figure 5, the differentiated cells secreted around 50 pmol/L of C-peptide at a low glucose concentration (5.5 mM) and approximately five as much at a high glucose concentration (25 mM).

### Effect on STZ rats blood sugar changes after cell transplantation

**Figure 6:**
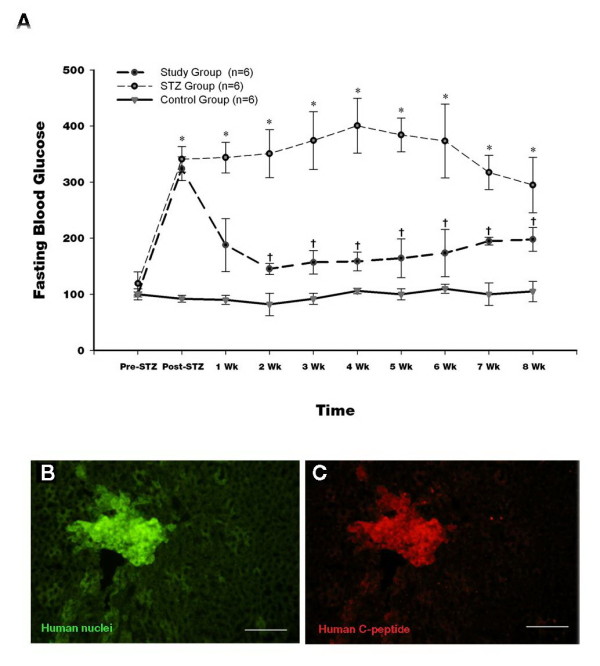
**(A) Changes in blood glucose levels in STZ-induced diabetic rats after transplantation of insulin-producing cells into the portal vein via the Port-A catheter (study group), non-transplanted STZ-induced diabetic rats (STZ group), and normal non-diabetic rats.** (*, P ≪ 0.05 compared to control group; †, P ≪ 0.05 compared to STZ group). Results are presented as the mean ± SEM for 6 rats. Double-immunostaining for human C-peptide and human nuclei in the liver of the STZ-induced diabetic rats at 8 weeks after transplantation of insulin-producing cells. **(B)** Stained with anti-human nuclei antibody (green). **(C)** Stained with anti-human C-peptide antibody (red). Bars = 100 μm.

The rats began to show hyperglycemia on the final day of STZ treatment. In the study group, blood sugar levels were reduced significantly 4 weeks after transplantation (P ≪ 0.05) (Figure 6A). The STZ group showed hyperglycemia.

### Immunofluorescent staining for human C-peptide and human cell nuclei of the rat livers after transplantation

By six weeks post-transplantation, insulin-producing cell function was evidenced by the appearance of both human C-peptide and human cell nuclei in the same location within the lobules of the rat liver (Figure 6B and 6C). Conversely, neither the human C-peptide or human cell nuclei were detected in the Sham group (data not shown). These findings suggest that the insulin-secreting cells differentiated from human Wharton’s jelly stem cells were able to function as islet-like structures following transplantation.

## Discussion

Adult bone marrow-derived cells can be induced to differentiate into insulin-producing cells under defined conditions [17]. Stem cell regeneration is an attractive insulin replacement therapy for those with insulin dependent DM. Stem cells from the pancreas [34,35], bone marrow [29], umbilical cord blood [25], and embryo [36] have previously been used in research on regeneration therapies for DM. Recently, we found that Wharton’s jelly from the human umbilical cord contains fibroblast-like cells, which are similar to MSCs [26]. In this study, we investigated the ability of these cells to differentiate into insulin-producing cells, as well as the potential curative effects of transplanting the insulin-producing cells into the livers of diabetic rats. Simultaneously, we tested the usefulness of the modified Port-A catheter in transplantation.

Our results illustrate that human umbilical cord MSCs could be differentiated into insulin-producing cells following incubation under specific conditions [17]. Based on current references of pancreas endocrine cell development, a combination of various factors, including activin A, sodium butyrate, growth factors in serum free media supplements were used in this study (Figure 1).

Due to the controversy surrounding insulin uptake by cells from media supplements [37,38], we used human C-peptide to characterize insulin production by our cells. Proinsulin, the precursor of insulin, is composed of 3 segments, the A-chain, B-chain, and C-peptide. Although C-peptide is released from proinsulin, unlike the A- and B-chains, it is not taken up by the cells. Thus, levels of C-peptide can be used as a marker of insulin secretion. After exposure of MSCs to differentiation conditions, immunocytochemical staining revealed that the cells expressed both insulin and C-peptide.

In a prior study we demonstrated that pancreatic endocrine precursor (PEP) cells could be generated from human umbilical cord MSCs [27]. In our *in vitro* studies, expression of β-cell development-related genes was examined by reverse transcriptase and real time PCR before and after induction of differentiation. After differentiation for 17 days, the insulin-producing cells expressed the following pancreatic β-cell development-related genes: *Pax4Nkx2.2MafANeuroDIsl-1Glut2* and *insulin*. Additionally, our C-peptide secretion assays revealed that the differentiated cells generated *in vitro* displayed functional characteristics of insulin-producing cells. After the cells were transplanted into the NOD mice via a retro-orbital vein, blood sugar levels tended to decrease [27].

In this study, Wharton’s jelly was induced to differentiate into islet-like cell aggregates. After differentiation for 10 days, the insulin-producing cells expressed the following pancreatic β-cell development-related genes: *Pax4*, *Pax1* and *insulin.* Additionally, we found greater expression of C-peptide in differentiated versus undifferentiated MSCs (Figure 1A). After the cells were transplanted into the STZ induced rats via a portal, we found that blood sugar levels tended to decrease in comparison to STZ rats receiving sham transplantation. This method of developing insulin-producing cells was more effective, inexpensive and less time consuming.

In the current study, Wharton’s jelly tissue was used as opposed to pancreatic stem cells, as the former contains much greater quantities of stem cells than the pancreatic duct. Specifically, each cubic centimeter of Wharton’s jelly sample contains 1–1.5 × 10^4^ MSCs and the number of cells increases 300-fold after seven passages, providing a plentiful supply of cells for transplantation. In addition, the use of Wharton’s jelly stem cells is preferable to embryonic stem (ES) cells, as doing so avoids the risk of teratoma formation as well as the ethical issues inherent in using ES cells.

The portal vein [39], renal subcapsular space [28], and tail vein [29] have been previously used as stem cell transplant locations for insulin regeneration therapies in the rat. In this study, cells were transplanted into the rat liver via the portal vein. Interestingly, we have observed that blood sugar levels tend to decrease sooner when using hepatic portal vein transplantation instead of renal subcapsular transplantation [28]. These results are likely related to the finding that transplantation into the liver is more advantageous than renal subcapsular transplantation for diabetes therapy, as the former technique provides a larger surface area for implantation and recapitulates an orthotopic site physiologically. Specifically, secreted insulin enters the portal system (via the superior mesenteric vein) rather than the systemic venous system (via the renal vein) [40]. In our previous study [27], transplantation of insulin-producing cells via the retro-orbital vein was used to treat NOD mice. Though techniquely it is easier to perform and less invasive than via portal vein, higher mortality rate in mice and less number (1 × 10^5^) of MSCs could be used were the major obstacles. Transplantation of MSCs via retro-orbital vein may only be used for the smaller animals. It has been reported that insulin-producing cells were injected directly into liver parenchymal of STZ induced diabetic rats to lower blood glucose level [41]. However, this transplantation method applied to clinical setting is limited. In clinical application, the Edmonton Protocol involves isolating islets from a cadaveric donor pancreas. Each recipient needs to transplant islets isolated from one to as many as three donors. The islets are infused into the patient’s portal vein, then these islets are stored at the liver to produces insulin. For clinical application to use hMSCs as a cell transplantation source for diabetic regeneration therapy, proof of usefulness of hMSCs transplantation via the portal vein in a large animal diabetic model is needed.

In order to test the function of the MSC-derived insulin-producing cells *in vivo*, we transplanted the differentiated cells into STZ-induced DM rats via a Port-A catheter into the portal vein. The modified Port-A catheter used in this study has two main advantages over techniques described in previous studies. First, since only a very small part of the catheter is inserted into the widest part of the portal vein, no veins are clamped permanently, in contrast to previous methods [42,43], and the disturbance to intestinal blood flow is minimized. Additionally, the use of a port enhances catheter longevity, thereby permitting longer periods of infusion. Indeed, the reported duration of use of a Port-A catheter is 9–34 months [44].

Following transplantation of insulin-producing cells into diabetic rats in the current study, C-peptide was found in the transplanted cells of the liver and blood glucose levels decreased. Both of these findings suggest that the transplanted cells secreted functional insulin. Indeed, on the fourth week after transplantation, blood glucose levels decreased to approximately 250 mg/dl in the compared to 530 mg/dl in the STZ controls. Nevertheless, we believe that transplantation may slow down the appearance of symptoms of DM rather than cure the disease.

## Conclusion

Our results show that human MSCs derived from umbilical cord can differentiate into pancreatic lineage cells *in vitro* and function as insulin-producing cells both *in vitro* and *in vivo*. Thus, these cells are a promising stem cell source for β-cell regeneration. Additionally, the modified Port-A catheter used in this study is an important method for the transplantation of insulin-producing cells. Further work is required to examine the curative effects on larger animal models and humans.

## Competing interests

The authors declare that they have no competing interests.

## Authors’ contributions

All authors participated in various aspects of the study analysis and interpretation of the data, and to the development of the report. The final version was seen and approved by all authors.
